# Supplementary material for: Parkinson’s Disease Pathogenic Variants: Cross-Ancestry Analysis and Microarray Data Validation
Source: medRxiv. 2024 Dec 17:2024.12.16.24319097. Preprint. [Version 1] doi: 10.1101/2024.12.16.24319097 (PMC11702716; doi:10.1101/2024.12.16.24319097)
Supplement: Supplement 2 [file media-2.pdf]

Demographics Overview

Ancestry	Total			Cases ("PD")			Control ("Control")			Case ("Other")		
	n	age(mean)	age(sd)	n	age(mean)	age(sd)	n	age(mean)	age(sd)	n	age(mean)	age(sd)
AAC	1,111	65.78	10.45	285	65.45	11.07	801	65.84	10.27	25	67.34	10.32
AFR	2,643	63.56	14.81	942	63.26	12.14	1,679	65.59	15.36	22	66.87	10.25
AJ	2,655	67.96	10.55	1,292	70.04	9.89	411	67.81	9.88	952	65.03	10.96
AMR	646	61.64	11.93	458	61.65	12.71	155	61.19	9.45	33	63.90	8.95
CAH	851	55.44	17.17	525	61.06	12.73	302	46.50	19.39	24	63.98	9.48
CAS	903	58.92	9.72	552	61.02	10.68	343	55.05	5.75	8	60.31	15.98
EAS	5,167	65.01	10.93	2,662	67.51	10.09	2,461	62.40	11.15	44	70.88	8.94
EUR	38,839	65.54	11.27	21,198	66.74	10.89	9,214	62.11	13.16	8,427	65.80	9.62
FIN	114	65.91	11.34	98	63.95	10.95	8	76.15	11.78	8	71.25	6.75
MDE	581	59.37	12.00	311	64.14	12.41	225	55.45	9.69	45	58.67	13.74
SAS	635	59.74	15.02	387	62.94	12.50	222	54.95	16.84	26	73.40	5.55
Total	54,145	65.12	11.64	28,710	66.54	11.03	15,821	61.83	13.37	9,614	65.7	9.82

PD: Parkinson's Disease

AAC: African American
AFR: African
AJ: Ashkenazi Jew
AMR: Admixed American/Latin American
CAH: Complex Admixture History
CAS: Central Asian
EAS: East Asian
EUR: European
FIN: Finnish
MDE: Middle Eastern
SAS: South Asian
