# Supplementary material for: Parkinson’s Disease Pathogenic Variants: Cross-Ancestry Analysis and Microarray Data Validation
Source: medRxiv. 2024 Dec 17:2024.12.16.24319097. Preprint. [Version 1] doi: 10.1101/2024.12.16.24319097 (PMC11702716; doi:10.1101/2024.12.16.24319097)
Supplement: Supplement 3 [file media-3.pdf]

ClinVar Annotations for the Pathogenic Variants within Established PD Genes

Chr	Start	End	Ref	Alt	Func.refGene	Gene.refGene	GeneDetail.refGene	ExonicFunc.refGene	AAChange.refGene	avsnp151	CLNALLELEID	CLNDN	CLNDISDB	CLNREVSTAT	CLNSIG	SIFT_score	SIFT converted rankscore	SIFT_pred
chr12	40310434	40310434	C	T	exonic	LRRK2	.	nonsynonymous SNV	LRRK2:NM_198578:exon31:c.C4321T:p.R1441C	rs33939927	16977	not_provided|Autosomal_dominant_Parkinson_disease_8	MedGen:C3661900|MONDO:MONDO	criteria_provided,_multiple_submitters,_no_conflicts	Pathogenic	0.117	0.282	T
chr15	89319225	89319225	T	A	intronic	POLG	.	.	.	rs778573169	580246	not_provided|Inborn_genetic_diseases|Progressive_sclerosing_poliodystrophy	MedGen:C3661900|MeSH:D030342,M	criteria_provided,_multiple_submitters,_no_conflicts	Pathogenic	.	.	.
chr15	89321780	89321780	G	A	exonic	POLG	.	nonsynonymous SNV	POLG:NM_001126131:exon16:c.C2554T:p.R852C,<br>POLG:NM_002693:exon16:c.C2554T:p.R852C	rs144500145	202960	Inborn_genetic_diseases|POLG-Related_Spectrum_Disorders|Intellectual_disability|not_provided|P	MeSH:D030342,MedGen:C0950123|M	criteria_provided,_multiple_submitters,_no_conflicts	Pathogenic	0	0.912	D
chr15	89321792	89321792	C	T	exonic	POLG	.	nonsynonymous SNV	POLG:NM_001126131:exon16:c.G2542A:p.G848S,<br>POLG:NM_002693:exon16:c.G2542A:p.G848S	rs113994098	28541	Progressive_sclerosing_poliodystrophy|Mitochondrial_DNA_depletion_syndrome_4b|Sensory_ataxi	MONDO:MONDO:0008758,MedGen:	criteria_provided,_multiple_submitters,_no_conflicts	Pathogenic	0	0.912	D
chr15	89325610	89325610	G	A	exonic	POLG	.	nonsynonymous SNV	POLG:NM_001126131:exon10:c.C1789T:p.R597W,<br>POLG:NM_002693:exon10:c.C1789T:p.R597W	rs139717885	374507	Progressive_sclerosing_poliodystrophy|not_provided|Sensory_ataxic_neuropathy,_dysarthria,_and_	MONDO:MONDO:0008758,MedGen:	criteria_provided,_multiple_submitters,_no_conflicts	Pathogenic	0.002	0.721	D
chr15	89325679	89325679	G	A	exonic	POLG	.	nonsynonymous SNV	POLG:NM_001126131:exon10:c.C1720T:p.R574W,<br>POLG:NM_002693:exon10:c.C1720T:p.R574W	rs774474723	1438803	Progressive_sclerosing_poliodystrophy|not_provided	MONDO:MONDO:0008758,MedGen:	criteria_provided,_multiple_submitters,_no_conflicts	Pathogenic	0.001	0.784	D
chr15	89327166	89327166	C	T	splicing	POLG	NM_001126131:exon7:c.1433+1G>A;<br>NM_002693:exon7:c.1433+1G>A	.	.	rs771623994	264590	Progressive_sclerosing_poliodystrophy|POLG-related_disorder|not_provided|Progressive_external_	MONDO:MONDO:0008758,MedGen:	criteria_provided,_multiple_submitters,_no_conflicts	Pathogenic	.	.	.
chr15	89327201	89327201	C	T	exonic	POLG	.	nonsynonymous SNV	POLG:NM_001126131:exon7:c.G1399A:p.A467T,<br>POLG:NM_002693:exon7:c.G1399A:p.A467T	rs113994095	28535	Progressive_sclerosing_poliodystrophy|Mitochondrial_DNA_depletion_syndrome_4b|Sensory_ataxi	MONDO:MONDO:0008758,MedGen:	criteria_provided,_multiple_submitters,_no_conflicts	Pathogenic	0.003	0.682	D
chr15	89329041	89329041	G	A	exonic	POLG	.	nonsynonymous SNV	POLG:NM_001126131:exon4:c.C925T:p.R309C,<br>POLG:NM_002693:exon4:c.C925T:p.R309C	rs886041592	264743	not_provided|Progressive_sclerosing_poliodystrophy|Progressive_external_ophthalmoplegia_with_	MedGen:C3661900|MONDO:MONDO	criteria_provided,_multiple_submitters,_no_conflicts	Pathogenic	0	0.912	D
chr1	155235196	155235196	G	A	exonic	GBA	.	nonsynonymous SNV	GBA:NM_001171811:exon9:c.C1243T:p.R415C,<br>GBA:NM_001171812:exon9:c.C1357T:p.R453C,<br>GBA:NM_000157:exon10:c.C1504T:p.R502C,<br>GBA:NM_001005741:exon11:c.C1504T:p.R502C,<br>GBA:NM_001005742:exon11:c.C1504T:p.R502C	rs80356771	19334	Parkinson_disease,_late-onset|Gaucher_disease-ophthalmoplegia-cardiovascular_calcification_syn	MONDO:MONDO:0008199,MedGen:	criteria_provided,_multiple_submitters,_no_conflicts	Pathogenic	0.001	0.784	D
chr1	155236295	155236295	G	A	exonic	GBA	.	nonsynonymous SNV	GBA:NM_001171811:exon7:c.C913T:p.R305W,<br>GBA:NM_001171812:exon7:c.C1027T:p.R343W,<br>GBA:NM_000157:exon8:c.C1174T:p.R392W,<br>GBA:NM_001005741:exon9:c.C1174T:p.R392W,<br>GBA:NM_001005742:exon9:c.C1174T:p.R392W	rs121908308	801582	Gaucher_disease_type_I|Gaucher_disease_type_II|Gaucher_disease_type_III|Gaucher_disease-o	MONDO:MONDO:0009265,MedGen:	criteria_provided,_single_submitter	Pathogenic	0.024	0.501	D
chr1	155237453	155237453	C	T	exonic	GBA	.	nonsynonymous SNV	GBA:NM_001171811:exon6:c.G626A:p.R209Q,<br>GBA:NM_001171812:exon6:c.G740A:p.R247Q,<br>GBA:NM_000157:exon7:c.G887A:p.R296Q,<br>GBA:NM_001005741:exon8:c.G887A:p.R296Q,<br>GBA:NM_001005742:exon8:c.G887A:p.R296Q	rs78973108	19367	Parkinson_disease,_late-onset|Gaucher_disease-ophthalmoplegia-cardiovascular_calcification_syn	MONDO:MONDO:0008199,MedGen:	criteria_provided,_multiple_submitters,_no_conflicts	Pathogenic	0.009	0.614	D
chr1	155238206	155238206	A	C	exonic	GBA	.	nonsynonymous SNV	GBA:NM_001171811:exon5:c.T428G:p.V143G,<br>GBA:NM_001171812:exon5:c.T542G:p.V181G,<br>GBA:NM_000157:exon6:c.T689G:p.V230G,<br>GBA:NM_001005741:exon7:c.T689G:p.V230G,<br>GBA:NM_001005742:exon7:c.T689G:p.V230G	rs381427	916795	Gaucher_disease	MONDO:MONDO:0018150,MedGen:	criteria_provided,_single_submitter	Pathogenic	0.01	0.564	D
chr1	155238215	155238215	T	C	exonic	GBA	.	nonsynonymous SNV	GBA:NM_001171811:exon5:c.A419G:p.N140S,<br>GBA:NM_001171812:exon5:c.A533G:p.N178S,<br>GBA:NM_000157:exon6:c.A680G:p.N227S,<br>GBA:NM_001005741:exon7:c.A680G:p.N227S,<br>GBA:NM_001005742:exon7:c.A680G:p.N227S	rs364897	19353	Gaucher_disease-ophthalmoplegia-cardiovascular_calcification_syndrome|Gaucher_disease_type_	MONDO:MONDO:0009268,MedGen:	criteria_provided,_multiple_submitters,_no_conflicts	Pathogenic	0.241	0.191	T
chr1	155238630	155238630	G	A	exonic	GBA	.	nonsynonymous SNV	GBA:NM_001171811:exon4:c.C214T:p.R72W,<br>GBA:NM_001171812:exon4:c.C328T:p.R110W,<br>GBA:NM_000157:exon5:c.C475T:p.R159W,<br>GBA:NM_001005741:exon6:c.C475T:p.R159W,<br>GBA:NM_001005742:exon6:c.C475T:p.R159W	rs439898	76478	Gaucher_disease|not_provided	MONDO:MONDO:0018150,MedGen:	criteria_provided,_multiple_submitters,_no_conflicts	Pathogenic	0	0.912	D
chr1	155239934	155239934	G	A	exonic	GBA	.	nonsynonymous SNV	GBA:NM_000157:exon3:c.C259T:p.R87W,<br>GBA:NM_001171812:exon3:c.C259T:p.R87W,<br>GBA:NM_001005741:exon4:c.C259T:p.R87W,<br>GBA:NM_001005742:exon4:c.C259T:p.R87W	rs1141814	19360	Parkinson_disease,_late-onset|Gaucher_disease-ophthalmoplegia-cardiovascular_calcification_syn	MONDO:MONDO:0008199,MedGen:	criteria_provided,_multiple_submitters,_no_conflicts	Pathogenic	0	0.912	D
chr1	16988455	16988455	C	T	exonic	ATP13A2	.	nonsynonymous SNV	ATP13A2:NM_001141974:exon23:c.G2497A:p.G833R<br>ATP13A2:NM_001141973:exon24:c.G2614A:p.G872R<br>ATP13A2:NM_022089:exon24:c.G2629A:p.G877R	rs144701072	77001	Kufor-Rakeb_syndrome	MONDO:MONDO:0011706,MedGen:	no_assertion_criteria_provided	Pathogenic	0	0.912	D
chr1	16989961	16989961	G	A	exonic	ATP13A2	.	stopgain	ATP13A2:NM_001141973:exon22:c.C2440T:p.R814X,<br>ATP13A2:NM_022089:exon22:c.C2455T:p.R819X	rs866035312	493540	not_provided|Autosomal_recessive_spastic_paraplegia_type_78|Kufor-Rakeb_syndrome	MedGen:C3661900|MONDO:MONDO	criteria_provided,_multiple_submitters,_no_conflicts	Pathogenic	.	.	.
chr1	16992345	16992345	G	A	exonic	ATP13A2	.	stopgain	ATP13A2:NM_001141973:exon18:c.C1888T:p.Q630X,<br>ATP13A2:NM_001141974:exon18:c.C1888T:p.Q630X,<br>ATP13A2:NM_022089:exon18:c.C1903T:p.Q635X	rs773246271	447471	Kufor-Rakeb_syndrome|Autosomal_recessive_spastic_paraplegia_type_78|not_provided	MONDO:MONDO:0011706,MedGen:	criteria_provided,_multiple_submitters,_no_conflicts	Pathogenic	.	.	.
chr1	16996059	16996059	G	A	exonic	ATP13A2	.	stopgain	ATP13A2:NM_001141973:exon15:c.C1444T:p.R482X,<br>ATP13A2:NM_001141974:exon15:c.C1444T:p.R482X,<br>ATP13A2:NM_022089:exon15:c.C1459T:p.R487X	rs1303653650	511191	Inborn_genetic_diseases|Kufor-Rakeb_syndrome|Autosomal_recessive_spastic_paraplegia_type_7	MeSH:D030342,MedGen:C0950123|M	criteria_provided,_multiple_submitters,_no_conflicts	Pathogenic	.	.	.
chr1	17005450	17005450	C	T	exonic	ATP13A2	.	stopgain	ATP13A2:NM_001141973:exon3:c.G212A:p.W71X,<br>ATP13A2:NM_001141974:exon3:c.G212A:p.W71X,<br>ATP13A2:NM_022089:exon3:c.G212A:p.W71X	rs373607247	425322	not_provided|Inborn_genetic_diseases	MedGen:CN517202|MeSH:D030342,M	criteria_provided,_multiple_submitters,_no_conflicts	Pathogenic	.	.	.
chr1	20645640	20645640	T	C	exonic	PINK1	.	nonsynonymous SNV	PINK1:NM_032409:exon5:c.T1040C:p.L347P	rs28940285	17447	Autosomal_recessive_early-onset_Parkinson_disease_6|not_provided	MONDO:MONDO:0011613,MedGen:	criteria_provided,_multiple_submitters,_no_conflicts	Pathogenic	0	0.912	D
chr1	20649109	20649109	C	T	exonic	PINK1	.	stopgain	PINK1:NM_032409:exon7:c.C1366T:p.Q456X	rs45539432	17454	not_provided|Autosomal_recessive_early-onset_Parkinson_disease_6	MedGen:C3661900|MONDO:MONDO	criteria_provided,_multiple_submitters,_no_conflicts	Pathogenic	.	.	.
chr1	20649217	20649217	C	T	exonic	PINK1	.	stopgain	PINK1:NM_032409:exon7:c.C1474T:p.R492X	rs34208370	425331	Autosomal_recessive_early-onset_Parkinson_disease_6|not_provided	MONDO:MONDO:0011613,MedGen:	criteria_provided,_multiple_submitters,_no_conflicts	Pathogenic	.	.	.
chr20	5100921	5100921	C	A	exonic	TMEM230	.	nonsynonymous SNV	TMEM230:NM_001009925:exon4:c.G233T:p.R78L,<br>TMEM230:NM_001330985:exon4:c.G233T:p.R78L,<br>TMEM230:NM_001330986:exon4:c.G233T:p.R78L,<br>TMEM230:NM_014145:exon4:c.G233T:p.R78L,<br>TMEM230:NM_001009923:exon5:c.G422T:p.R141L,<br>TMEM230:NM_001009924:exon5:c.G233T:p.R78L,<br>TMEM230:NM_001330984:exon5:c.G233T:p.R78L	rs764786986	244095	not_provided	MedGen:C3661900	criteria_provided,_single_submitter	Pathogenic	0.005	0.654	D
chr21	32726907	32726907	C	A	exonic	SYNJ1	.	stopgain	SYNJ1:NM_003895:exon2:c.G106T:p.E36X	rs1040540690	1393547	Developmental_and_epileptic_encephalopathy,_53|Early-onset_Parkinson_disease_20	MONDO:MONDO:0033362,MedGen:	criteria_provided,_single_submitter	Pathogenic	.	.	.
chr22	38115658	38115658	G	A	exonic	PLA2G6	.	stopgain	PLA2G6:NM_001004426:exon13:c.C1741T:p.R581X,<br>PLA2G6:NM_001199562:exon13:c.C1741T:p.R581X,<br>PLA2G6:NM_001349865:exon13:c.C1741T:p.R581X,<br>PLA2G6:NM_001349866:exon13:c.C1741T:p.R581X,<br>PLA2G6:NM_001349868:exon13:c.C1225T:p.R409X,<br>PLA2G6:NM_001349864:exon14:c.C1903T:p.R635X,<br>PLA2G6:NM_001349869:exon14:c.C1207T:p.R403X,<br>PLA2G6:NM_003560:exon14:c.C1903T:p.R635X,<br>PLA2G6:NM_001349867:exon15:c.C1369T:p.R457X	rs587784339	169779	Infantile_neuroaxonal_dystrophy|PLA2G6-associated_neurodegeneration|not_provided|Iron_accum	MONDO:MONDO:0024457,MedGen:	criteria_provided,_multiple_submitters,_no_conflicts	Pathogenic	.	.	.
chr22	38132917	38132917	C	A	exonic	PLA2G6	.	nonsynonymous SNV	PLA2G6:NM_001349868:exon6:c.G313T:p.D105Y,<br>PLA2G6:NM_001004426:exon7:c.G991T:p.D331Y,<br>PLA2G6:NM_001199562:exon7:c.G991T:p.D331Y,<br>PLA2G6:NM_001349864:exon7:c.G991T:p.D331Y,<br>PLA2G6:NM_001349865:exon7:c.G991T:p.D331Y,<br>PLA2G6:NM_001349866:exon7:c.G991T:p.D331Y,<br>PLA2G6:NM_003560:exon7:c.G991T:p.D331Y,<br>PLA2G6:NM_001349867:exon8:c.G457T:p.D153Y,<br>PLA2G6:NM_001349869:exon8:c.G457T:p.D153Y	rs199935023	39328	Infantile_neuroaxonal_dystrophy|Autosomal_recessive_Parkinson_disease_14|PLA2G6-associated	MONDO:MONDO:0024457,MedGen:	criteria_provided,_multiple_submitters,_no_conflicts	Pathogenic	0.003	0.682	D
chr22	38169318	38169318	G	A	exonic	PLA2G6	.	stopgain	PLA2G6:NM_001004426:exon2:c.C109T:p.R37X,<br>PLA2G6:NM_001199562:exon2:c.C109T:p.R37X,<br>PLA2G6:NM_001349864:exon2:c.C109T:p.R37X,<br>PLA2G6:NM_001349865:exon2:c.C109T:p.R37X,<br>PLA2G6:NM_001349866:exon2:c.C109T:p.R37X,<br>PLA2G6:NM_003560:exon2:c.C109T:p.R37X	rs200075782	39327	Iron_accumulation_in_brain|Neurodegeneration_with_brain_iron_accumulation_2B|Infantile_neuroa	Human_Phenotype_Ontology:HP:001	criteria_provided,_multiple_submitters,_no_conflicts	Pathogenic	.	.	.
chr6	161350214	161350214	G	C	intronic	PRKN	.	.	.	rs765860776	1867959	not_provided	MedGen:C3661900	criteria_provided,_single_submitter	Pathogenic	.	.	.
chr6	161785793	161785793	C	G	exonic	PRKN	.	nonsynonymous SNV	PRKN:NM_013988:exon4:c.G403C:p.G135R,<br>PRKN:NM_013987:exon6:c.G766C:p.G256R,<br>PRKN:NM_004562:exon7:c.G850C:p.G284R	rs751037529	395419	Autism_spectrum_disorder|not_provided|Autosomal_recessive_juvenile_Parkinson_disease_2	MONDO:MONDO:0005258,MeSH:D0	criteria_provided,_single_submitter	Pathogenic	0	0.912	D
chr6	161785839	161785839	A	T	exonic	PRKN	.	stopgain	PRKN:NM_013988:exon4:c.T357A:p.C119X,<br>PRKN:NM_013987:exon6:c.T720A:p.C240X,<br>PRKN:NM_004562:exon7:c.T804A:p.C268X	rs377554392	793178	not_provided	MedGen:C3661900	criteria_provided,_multiple_submitters,_no_conflicts	Pathogenic	.	.	.
chr6	162443314	162443314	A	T	exonic	PRKN	.	nonsynonymous SNV	PRKN:NM_004562:exon2:c.T167A:p.V56E,<br>PRKN:NM_013987:exon2:c.T167A:p.V56E,<br>PRKN:NM_013988:exon2:c.T167A:p.V56E	rs137853059	22086	not_provided|Autosomal_recessive_juvenile_Parkinson_disease_2	MedGen:CN517202|MONDO:MONDO	criteria_provided,_multiple_submitters,_no_conflicts	Pathogenic	0.001	0.784	D
chr6	162443356	162443356	C	G	exonic	PRKN	.	nonsynonymous SNV	PRKN:NM_004562:exon2:c.G125C:p.R42P,<br>PRKN:NM_013987:exon2:c.G125C:p.R42P,<br>PRKN:NM_013988:exon2:c.G125C:p.R42P	rs368134308	634774	not_provided	MedGen:C3661900	criteria_provided,_single_submitter	Pathogenic	0.011	0.564	D

Polyphen2 HDIV score	Polyphen2 HDIV rankscore	Polyphen2 HDIV pred	Polyphen2 HVAR score	Polyphen2 HVAR rankscore	Polyphen2 HVAR pred	LRT score	LRT converted rankscore	LRT pred	MutationTaster score	MutationTaster converted rankscore	MutationTaster pred	MutationAssessor score	MutationAssessor score rankscore	MutationAssessor pred	FATHMM score	FATHMM converted rankscore	FATHMM pred	PROVEAN score	PROVEAN converted rankscore	PROVEAN pred	VEST3 score	VEST3 rankscore	MetaSVM score	MetaSVM rankscore	MetaSVM pred	MetaLR score	MetaLR rankscore	MetaLR pred	M-CAP score	M-CAP rankscore	M-CAP pred	CADD raw	CADD raw rankscore	CADD phred	DANN score	DANN rankscore	fathmm-MKL coding score	fathmm-MKL coding rankscore	fathmm-MKL coding pred
1	0.899	D	0.994	0.875	D	0.001	0.424	D	1	0.473	D	2.455	0.715	M	-1.38	0.803	T	-4.5	0.781	D	0.924	0.916	0.245	0.867	D	0.629	0.87	D	0.19	0.862	D	5.652	0.77	26.7	0.999	0.994	0.776	0.379	D
.	.	.	.	.	.	.	.	.	.	.	.	.	.	.	.	.	.	.	.	.	.	.	.	.	.	.	.	.	.	.	.	.	.	.	.	.	.	.	.
1	0.899	D	0.999	0.916	D	0	0.843	D	1	0.81	D	3.54	0.933	H	-5	0.985	D	-7.91	0.962	D	0.988	0.992	1.091	0.993	D	0.973	0.991	D	0.542	0.958	D	7.949	0.956	35	0.999	0.998	0.997	0.986	D
1	0.899	D	1	0.971	D	0	0.843	D	1	0.81	A	3.545	0.934	H	-5.3	0.99	D	-5.93	0.89	D	0.987	0.991	1.065	0.985	D	0.979	0.993	D	0.546	0.958	D	7.354	0.949	34	0.999	0.944	0.994	0.96	D
1	0.899	D	0.998	0.875	D	0	0.843	D	1	0.588	D	2.65	0.778	M	-4.51	0.977	D	-5.83	0.883	D	0.939	0.933	1.091	0.993	D	0.954	0.985	D	0.327	0.917	D	7.163	0.944	34	0.999	0.989	0.871	0.463	D
1	0.899	D	0.998	0.875	D	0	0.843	D	1	0.81	D	2.845	0.829	M	-2.27	0.874	D	-6.39	0.911	D	0.921	0.913	0.969	0.967	D	0.876	0.959	D	0.328	0.917	D	7.906	0.956	35	0.999	0.989	0.985	0.838	D
.	.	.	.	.	.	.	.	.	1	0.81	D	.	.	.	.	.	.	.	.	.	.	.	.	.	.	.	.	.	.	.	.	5.333	0.722	25.8	0.996	0.717	0.918	0.541	D
1	0.899	D	0.996	0.832	D	0	0.843	D	1	0.81	A	1.95	0.526	M	-3.1	0.927	D	-3.27	0.655	D	0.91	0.9	0.779	0.942	D	0.832	0.944	D	0.215	0.875	D	6.527	0.899	31	0.999	0.996	0.99	0.902	D
1	0.899	D	0.997	0.85	D	0	0.843	D	1	0.81	D	3.555	0.935	H	-3.39	0.942	D	-6.42	0.912	D	0.972	0.973	1.039	0.979	D	0.925	0.975	D	0.226	0.881	D	7.881	0.956	35	0.999	0.995	0.988	0.872	D
0.999	0.764	D	0.932	0.65	D	0.003	0.361	N	1	0.588	A	2.705	0.794	M	-5.84	0.994	D	-5.28	0.843	D	0.903	0.893	1.058	0.983	D	0.968	0.99	D	0.917	0.994	D	6.394	0.883	29.6	0.999	0.966	0.829	0.418	D
0.999	0.899	D	0.97	0.764	D	0	0.457	D	0.968	0.423	D	2.67	0.784	M	-5.72	0.993	D	-2.13	0.5	N	0.569	0.778	1.088	0.992	D	0.968	0.99	D	0.886	0.991	D	5.422	0.735	26	0.999	0.982	0.7	0.341	D
1	0.899	D	0.995	0.832	D	0	0.629	D	1	0.513	A	3.29	0.905	M	-6.24	0.996	D	-3.57	0.705	D	0.896	0.885	1.063	0.984	D	0.984	0.995	D	0.89	0.992	D	6.932	0.933	33	0.999	0.999	0.964	0.685	D
0.001	0.147	B	0.001	0.154	B	0.006	0.321	N	1	0.588	D	1.215	0.307	L	-5.8	0.994	D	-3.81	0.718	D	0.915	0.906	0.508	0.907	D	0.816	0.938	D	0.422	0.938	D	-0.512	0.055	0.206	0.639	0.071	0.913	0.53	D
0.001	0.102	B	0.019	0.176	B	0	0.537	N	1	0.479	A	1.56	0.396	L	-5.71	0.993	D	-3.05	0.664	D	0.366	0.427	0.425	0.895	D	0.843	0.948	D	0.241	0.887	D	-1.034	0.031	0.013	0.46	0.036	0.816	0.407	D
0.969	0.764	D	0.874	0.668	P	0	0.629	D	1	0.588	D	3.67	0.945	H	-7.03	0.998	D	-6.84	0.93	D	0.911	0.906	0.979	0.968	D	0.993	0.998	D	0.807	0.985	D	6.19	0.854	28.6	0.998	0.894	0.966	0.697	D
1	0.899	D	1	0.971	D	0	0.457	N	1	0.81	D	2.91	0.844	M	-6.3	0.996	D	-6.88	0.932	D	0.885	0.873	1.085	0.99	D	0.975	0.992	D	0.885	0.991	D	4.813	0.646	24.8	0.997	0.774	0.873	0.466	D
1	0.899	D	1	0.971	D	0	0.843	D	1	0.537	D	4.445	0.988	H	-2.62	1	D	-7.63	0.964	D	0.984	0.987	0.913	0.959	D	0.998	1	D	0.869	0.99	D	7.438	0.951	34	0.999	0.979	0.993	0.936	D
.	.	.	.	.	.	0.491	0.121	N	1	0.81	A	.	.	.	.	.	.	.	.	.	.	.	.	.	.	.	.	.	.	.	.	9.496	0.963	35	0.996	0.75	0.089	0.146	N
.	.	.	.	.	.	0	0.843	D	1	0.81	A	.	.	.	.	.	.	.	.	.	.	.	.	.	.	.	.	.	.	.	.	11.471	0.975	37	0.998	0.85	0.998	0.993	D
.	.	.	.	.	.	0	0.843	D	1	0.81	A	.	.	.	.	.	.	.	.	.	.	.	.	.	.	.	.	.	.	.	.	10.346	0.967	36	0.997	0.833	0.943	0.606	D
.	.	.	.	.	.	0	0.481	D	1	0.81	A	.	.	.	.	.	.	.	.	.	.	.	.	.	.	.	.	.	.	.	.	10.303	0.967	36	0.993	0.599	0.777	0.379	D
1	0.899	D	0.999	0.971	D	0	0.843	D	1	0.81	A	3.84	0.958	H	-1.18	0.783	T	-6.67	0.923	D	0.94	0.934	0.775	0.941	D	0.739	0.911	D	0.299	0.909	D	5.08	0.684	25.3	0.998	0.914	0.97	0.718	D
.	.	.	.	.	.	0.145	0.181	N	1	0.81	A	.	.	.	.	.	.	.	.	.	.	.	.	.	.	.	.	.	.	.	.	12.745	0.986	40	0.997	0.822	0.79	0.388	D
.	.	.	.	.	.	0	0.843	D	1	0.81	D	.	.	.	.	.	.	.	.	.	.	.	.	.	.	.	.	.	.	.	.	15.572	0.999	51	0.998	0.848	0.931	0.571	D
0.89	0.622	P	0.716	0.608	P	0	0.629	D	1	0.81	D	3.005	0.862	M	0.8	0.488	T	-5.58	0.864	D	0.889	0.877	-0.396	0.722	T	0.291	0.663	T	0.039	0.586	D	5.927	0.814	27.6	0.998	0.882	0.853	0.441	D
.	.	.	.	.	.	.	.	.	1	0.81	A	.	.	.	.	.	.	.	.	.	.	.	.	.	.	.	.	.	.	.	.	10.948	0.971	37	0.994	0.629	0.615	0.312	D
.	.	.	.	.	.	0	0.559	D	1	0.81	A	.	.	.	.	.	.	.	.	.	.	.	.	.	.	.	.	.	.	.	.	14.038	0.995	44	0.998	0.899	0.992	0.933	D
1	0.899	D	0.995	0.818	D	0	0.559	D	1	0.588	D	2.195	0.62	M	-0.3	0.679	T	-4.43	0.775	D	0.879	0.867	0.147	0.85	D	0.536	0.829	D	0.228	0.882	D	7.28	0.948	34	0.996	0.728	0.989	0.876	D
.	.	.	.	.	.	0.795	0.094	U	1	0.81	A	.	.	.	.	.	.	.	.	.	.	.	.	.	.	.	.	.	.	.	.	8.185	0.957	35	0.998	0.879	0.113	0.164	N
.	.	.	.	.	.	.	.	.	.	.	.	.	.	.	.	.	.	.	.	.	.	.	.	.	.	.	.	.	.	.	.	.	.	.	.	.	.	.	.
1	0.899	D	1	0.971	D	0	0.843	D	1	0.499	D	2.44	0.71	M	-7	0.998	D	-6.9	0.944	D	0.966	0.969	0.976	0.968	D	0.992	0.998	D	0.494	0.951	D	6.786	0.924	32	0.999	0.998	0.934	0.58	D
.	.	.	.	.	.	0	0.843	D	1	0.81	A	.	.	.	.	.	.	.	.	.	.	.	.	.	.	.	.	.	.	.	.	9.852	0.964	36	0.986	0.429	0.312	0.241	N
0.987	0.715	D	0.88	0.728	P	0.002	0.369	N	0.977	0.81	A	2.33	0.67	M	-4.02	0.964	D	-4.3	0.789	D	0.94	0.934	1.06	0.983	D	0.918	0.973	D	0.402	0.934	D	6.194	0.855	28.6	0.988	0.461	0.919	0.542	D
0.998	0.715	D	0.96	0.719	D	0	0.843	D	0.988	0.81	D	3.335	0.911	M	-1.19	0.784	T	-3.86	0.752	D	0.968	0.968	0.502	0.906	D	0.674	0.887	D	0.124	0.806	D	7.095	0.941	33	0.998	0.868	0.808	0.401	D

Eigen coding or noncoding	Eigen-raw	Eigen-PC raw	GenoCanyon score	GenoCanyonscore rankscore	integrated fitCons score	integrated fitCons score rankscore	integrated confidence value	GERP++ RS	GERP++ RS rankscore	phyloP100way vertebrate	phyloP100way vertebrate rankscore	phyloP20way mammalian	phyloP20way mammalian rankscore	phastCons100way vertebrate	phastCons100way vertebrate rankscore	phastCons20way mammalian	phastCons20way mammalia rankscore	SiPhy 29way logOdds	SiPhy 29way logOdds rankscore	Interpro domain
c	0.361	0.259	0.999	0.396	0.554	0.246	0	4.74	0.596	3.725	0.544	0.852	0.362	0.998	0.411	0.885	0.372	15.915	0.792	Mitochondrial Rho-like|P-loop containing nucleoside triphosphate hydrolase|Roc domain|Small GTP-binding protein domain
.	.	.	.	.	.	.	.	.	.	.	.	.	.	.	.	.	.	.	.	.
c	1.009	0.949	1	0.983	0.672	0.522	0	5.5	0.813	9.508	0.97	1.048	0.713	1	0.715	0.994	0.587	19.399	0.946	DNA-directed DNA polymerase, family A, palm domain
c	0.811	0.719	1	0.747	0.672	0.522	0	4.56	0.555	7.498	0.802	0.935	0.49	1	0.715	0.995	0.604	15.416	0.746	DNA-directed DNA polymerase, family A, palm domain
c	0.589	0.504	1	0.747	0.707	0.73	0	4.05	0.462	2.763	0.47	1.048	0.713	1	0.715	1	0.888	13.225	0.592	.
c	0.691	0.674	1	0.983	0.707	0.73	0	4.99	0.658	8.04	0.891	1.048	0.713	1	0.715	1	0.888	18.267	0.899	.
c	0.997	0.824	1	0.983	0.284	0.042	0	4.99	0.658	7.271	0.777	0.892	0.403	1	0.715	0.888	0.373	18.463	0.907	.
c	0.662	0.641	1	0.983	0.707	0.73	0	5	0.661	7.468	0.798	0.892	0.403	1	0.715	0.98	0.49	18.482	0.907	.
c	1.071	1.022	1	0.983	0.707	0.73	0	5.84	0.934	10.003	0.997	1.048	0.713	1	0.715	1	0.888	20.135	0.98	Ribonuclease H-like domain
c	0.306	0.17	0.273	0.189	0.706	0.609	0	2.11	0.262	1.048	0.298	0.803	0.325	1	0.715	0.722	0.315	9.221	0.364	Glycoside hydrolase superfamily|Glycoside hydrolase, catalytic domain;Glycosyl hydrolase, family 13, all-beta
c	0.4	0.293	0.988	0.314	0.707	0.73	0	3.67	0.411	2.224	0.423	0.914	0.427	0.808	0.296	0.988	0.529	11.005	0.467	Glycoside hydrolase superfamily|Glycoside hydrolase, catalytic domain
c	0.638	0.512	1	0.489	0.706	0.609	0	3.51	0.391	5.235	0.65	0.818	0.335	1	0.715	0.999	0.75	10.668	0.447	Glycoside hydrolase superfamily|Glycoside hydrolase, catalytic domain
c	-0.862	-0.747	0.985	0.308	0.706	0.609	0	2.5	0.292	4.227	0.582	1.053	0.755	0.995	0.385	0.712	0.312	7.355	0.257	Glycoside hydrolase superfamily|Glycoside hydrolase, catalytic domain
c	-0.761	-0.71	0.859	0.252	0.706	0.609	0	1.25	0.204	3.36	0.518	0.935	0.49	1	0.715	0.804	0.337	7.407	0.26	Glycoside hydrolase superfamily|Glycoside hydrolase, catalytic domain
c	0.57	0.412	0.282	0.19	0.706	0.609	0	3.55	0.396	4.353	0.589	0.051	0.162	1	0.715	0.99	0.544	10.764	0.453	Glycoside hydrolase superfamily|Glycoside hydrolase, catalytic domain;Glycoside hydrolase, catalytic domain
c	0.001	-0.215	1	0.411	0.732	0.924	0	1.33	0.209	2.842	0.477	0.048	0.16	1	0.715	0.025	0.141	3.812	0.083	Glycosyl hydrolase, family 13, all-beta
c	0.818	0.661	1	0.5	0.713	0.817	0	3.46	0.386	7.512	0.805	0.855	0.374	1	0.715	0.997	0.653	9.609	0.386	HAD-like domain
c	0.249	-0.059	0.977	0.297	0.706	0.609	0	2.53	0.295	-0.032	0.121	0.953	0.551	0	0.063	0.109	0.192	8.173	0.302	.
c	1.031	0.869	1	0.747	0.706	0.609	0	5.73	0.897	9.905	0.986	0.994	0.605	1	0.715	0.988	0.529	18.453	0.906	P-type ATPase, cytoplasmic domain N
c	0.556	0.36	0.371	0.199	0.706	0.609	0	4.27	0.498	1.185	0.315	0.994	0.605	0.922	0.318	0.984	0.507	11.753	0.509	P-type ATPase, A domain
c	0.704	0.546	0.983	0.305	0.707	0.73	0	3.44	0.383	3.246	0.509	0.824	0.337	1	0.715	0.985	0.512	9.358	0.372	.
c	0.734	0.578	0.999	0.374	0.707	0.73	0	4.9	0.635	6.966	0.757	1.058	0.762	1	0.715	0.043	0.159	10.397	0.432	Protein kinase domain|Protein kinase-like domain
c	0.735	0.59	0.95	0.278	0.672	0.522	0	5.13	0.696	2.193	0.42	0.935	0.49	0.861	0.304	0.955	0.433	13.364	0.599	Protein kinase domain|Protein kinase-like domain
c	0.894	0.8	0.851	0.25	0.672	0.522	0	6.17	0.997	2.897	0.481	0.935	0.49	1	0.715	1	0.888	16.38	0.832	Protein kinase domain|Protein kinase-like domain
c	0.578	0.509	1	0.747	0.778	0.996	0	3.87	0.437	4.33	0.588	0.847	0.346	0.999	0.424	0.998	0.697	11.896	0.518	.
c	0.866	0.681	1	0.747	0.493	0.174	0	3.58	0.4	2.509	0.449	0.934	0.45	0.922	0.318	1	0.888	12.369	0.544	.
c	0.867	0.724	1	0.747	0.706	0.609	0	4.53	0.548	7.506	0.804	0.953	0.551	1	0.715	0.998	0.697	17.634	0.88	Acyl transferase/acyl hydrolase/lysophospholipase|Patatin/Phospholipase A2-related
c	0.798	0.781	1	0.747	0.707	0.73	0	5.49	0.809	7.358	0.786	0.935	0.49	1	0.715	0.983	0.502	19.374	0.945	Ankyrin repeat-containing domain
c	0.355	0.066	1	0.48	0.706	0.609	0	4.63	0.57	1.884	0.391	0.953	0.551	0.185	0.239	0.29	0.235	14.39	0.664	.
.	.	.	.	.	.	.	.	.	.	.	.	.	.	.	.	.	.	.	.	.
c	0.766	0.697	1	0.431	0.554	0.246	0	5.75	0.904	6.982	0.758	0.935	0.49	1	0.715	0.923	0.397	17.711	0.882	.
c	-0.131	-0.413	0	0.065	0.554	0.246	0	-6.67	0.016	-0.202	0.094	0.234	0.26	0.533	0.27	0.999	0.75	15.684	0.771	.
c	0.732	0.732	1	0.489	0.516	0.203	0	5.63	0.861	7.205	0.772	1.199	0.96	1	0.715	0.995	0.604	16.131	0.812	Ubiquitin domain|Ubiquitin-related domain
c	0.785	0.709	0.994	0.336	0.554	0.246	0	4.74	0.596	0.748	0.258	0.935	0.49	0.996	0.391	1	0.888	15.471	0.751	Ubiquitin domain|Ubiquitin-related domain
