# Supplementary material for: Parkinson’s Disease Pathogenic Variants: Cross-Ancestry Analysis and Microarray Data Validation
Source: medRxiv. 2024 Dec 17:2024.12.16.24319097. Preprint. [Version 1] doi: 10.1101/2024.12.16.24319097 (PMC11702716; doi:10.1101/2024.12.16.24319097)
Supplement: Supplement 4 [file media-4.pdf]

Typed Pathogenic Variant Cluster Plot Classification

Table 3A

SNP	rsID	Gene	Classification	Number of NC	GP2 r7 Missing	GP2 r7 MAF
chr1:16988455:C:T	rs144701072	ATP13A2	Bad	20	0.017351	0.001302
chr1:16989961:G:A	rs866035312	ATP13A2	Bad	11	0.017162	0.00416
chr1:20645640:T:C	rs28940285	PINK1	Bad	10	0.003482	0.000517
chr1:20649109:C:T	rs45539432	PINK1	Good	0	0.006364	0.001309
chr1:20649217:C:T	rs34208370	PINK1	Bad	47	0.017892	0.003796
chr1:155235196:G:A	rs80356771	GBA	Good	1	0.002296	0.00132
chr1:155237453:C:T	rs78973108	GBA	Good	2	0.003762	0.001097
chr1:155238206:A:C	rs381427	GBA	Bad	23	0.008074	0.000208
chr1:155238215:T:C	rs364897	GBA	Good	0	0.001512	0.000275
chr6:161785793:C:G	rs751037529	PRKN	Good	0	0.002405	0.000103
chr6:161785839:A:T	rs377554392	PRKN	Good	1	0.001941	0.000103
chr6:162443314:A:T	rs137853059	PRKN	Good	0	0.00219	0.00025
chr6:161350214:G:C	rs765860776	PRKN	Good	4	0.003882	0.000759
chr6:162443356:C:G	rs368134308	PRKN	Good	1	0.002835	0.00019
chr12:40310434:C:T	rs33939927	LRRK2	Good	4	0.003917	0.001251
chr15:89321780:G:A	rs144500145	POLG	Bad	66	0.017689	0.004101
chr15:89321792:C:T	rs113994098	POLG	Bad	45	0.016234	0.002766
chr15:89325610:G:A	rs139717885	POLG	Good	1	0.004681	0.000889
chr15:89325679:G:A	rs774474723	POLG	Medium	3	0.004854	0.000691
chr15:89327166:C:T	rs771623994	POLG	Medium	8	0.006459	0.001431
chr15:89327201:C:T	rs113994095	POLG	Bad	13	0.008018	0.002619
chr15:89329041:G:A	rs886041592	POLG	Medium	4	0.009293	0.002384
chr22:38115658:G:A	rs587784339	PLA2G6	Bad	98	0.02223	0.001729
chr22:38132917:C:A	rs199935023	PLA2G6	Good	4	0.005746	0.000307
chr22:38169318:G:A	rs200075782	PLA2G6	Medium	6	0.006914	0.002151

Table 3B

Classification	N	Mean NC	Min NC	Max NC	Avg. Missingne	Avg. MAF
Good	12	1.5	0	4	0.003461	0.000654
Medium	4	5.25	3	8	0.00688	0.001664
Bad	9	37	10	98	0.014236	0.002355
