# Supplementary material for: Parkinson’s Disease Pathogenic Variants: Cross-Ancestry Analysis and Microarray Data Validation
Source: medRxiv. 2024 Dec 17:2024.12.16.24319097. Preprint. [Version 1] doi: 10.1101/2024.12.16.24319097 (PMC11702716; doi:10.1101/2024.12.16.24319097)
Supplement: Supplement 5 [file media-5.pdf]

gnomAD (v4.1.0) pathogenic variant information for Parkinson's Disease genes

gene	gnomAD_ID	Chromosome	Position	rsIDs	Reference	Alternate	ClinVar_ClnSignificance	ClinVar_varID	allele_count	allele_number	allele_freq	in_gp2
ATP13A2	1-16986881-CAG-C	1	16986881	rs1570759415	CAG	C	Pathogenic	660925	1	1614048	6.20E-07	FALSE
ATP13A2	1-16986885-GAGA-G	1	16986885	rs1057519290	GAGA	G	Pathogenic	374887	1	1614104	6.20E-07	FALSE
ATP13A2	1-16986886-A-AG	1	16986886	rs747617559	A	AG	Pathogenic	1968613	2	1614040	1.24E-06	FALSE
ATP13A2	1-16988455-C-T	1	16988455	rs144701072	C	T	Pathogenic	66099	15	1613774	9.29E-06	TRUE
ATP13A2	1-16989739-A-C	1	16989739	rs587777053	A	C	Pathogenic	66098	1	1614078	6.20E-07	FALSE
ATP13A2	1-16989961-G-A	1	16989961	rs866035312	G	A	Pathogenic	502116	9	1607028	5.60E-06	TRUE
ATP13A2	1-16992022-G-A	1	16992022	rs774115028	G	A	Pathogenic	1996186	1	1612960	6.20E-07	FALSE
ATP13A2	1-16992315-CT-C	1	16992315	rs2076962370	CT	C	Pathogenic	2923611	2	1612322	1.24E-06	FALSE
ATP13A2	1-16992345-G-A	1	16992345	rs773246271	G	A	Pathogenic	465252	46	1613016	2.85E-05	TRUE
ATP13A2	1-16992506-C-A	1	16992506		C	A	Pathogenic	1999337	1	1614160	6.20E-07	FALSE
ATP13A2	1-16996059-G-A	1	16996059	rs1303653650	G	A	Pathogenic	520750	20	1613950	1.24E-05	TRUE
ATP13A2	1-16996262-G-A	1	16996262	rs1057519293	G	A	Pathogenic	374890	15	1614168	9.29E-06	FALSE
ATP13A2	1-16997101-GC-G	1	16997101	rs1377055875	GC	G	Pathogenic	1385899	3	1613740	1.86E-06	FALSE
ATP13A2	1-16997112-G-GTC	1	16997112	rs762033589	G	GTC	Pathogenic	30833	1	1613832	6.20E-07	FALSE
ATP13A2	1-17000466-C-T	1	17000466	rs1334843918	C	T	Pathogenic	2046647	1	1613948	6.20E-07	FALSE
ATP13A2	1-17002312-G-A	1	17002312	rs1483668823	G	A	Pathogenic	1442231	1	1613710	6.20E-07	FALSE
ATP13A2	1-17002326-TG-T	1	17002326	rs758150853	TG	T	Pathogenic	1751269	7	1613436	4.34E-06	FALSE
ATP13A2	1-17004817-C-A	1	17004817	rs756152157	C	A	Pathogenic	3336424	5	1613772	3.10E-06	FALSE
ATP13A2	1-17005444-AC-A	1	17005444	rs1389678247	AC	A	Pathogenic	2931003	4	1613712	2.48E-06	FALSE
ATP13A2	1-17005449-C-T	1	17005449		C	T	Pathogenic	2419737	1	1614170	6.20E-07	FALSE
ATP13A2	1-17005450-C-T	1	17005450	rs373607247	C	T	Pathogenic	432661	14	1614062	8.67E-06	TRUE
FBXO7	22-32475003-A-C	22	32475003	rs753392528	A	C	Pathogenic	2887235	18	1536094	1.17E-05	FALSE
FBXO7	22-32475004-T-G	22	32475004	rs945794813	T	G	Pathogenic	2916108	10	1536252	6.51E-06	FALSE
FBXO7	22-32475004-T-A	22	32475004		T	A	Pathogenic	2960687	1	1536252	6.51E-07	FALSE
FBXO7	22-32475067-C-T	22	32475067	rs121918305	C	T	Pathogenic	4811	7	1546034	4.53E-06	FALSE
FBXO7	22-32478991-C-T	22	32478991	rs1370252127	C	T	Pathogenic	2661902	17	1613960	1.05E-05	FALSE
FBXO7	22-32479008-GA-G	22	32479008	rs1228608709	GA	G	Pathogenic	488517	4	1614138	2.48E-06	FALSE
FBXO7	22-32479126-TCC-T	22	32479126	rs750398883	TCC	T	Pathogenic	2150213	21	1614180	1.30E-05	FALSE
FBXO7	22-32479233-AG-A	22	32479233	rs2057448454	AG	A	Pathogenic	2884317	3	1614158	1.86E-06	FALSE
FBXO7	22-32483976-C-G	22	32483976	rs779737534	C	G	Pathogenic	2751184	1	1614208	6.19E-07	FALSE
FBXO7	22-32484021-A-AT	22	32484021		A	AT	Pathogenic	2757154	2	1614194	1.24E-06	FALSE
FBXO7	22-32484052-CTG-C	22	32484052	rs778770873	CTG	C	Pathogenic	2699868	1	1614176	6.20E-07	FALSE
FBXO7	22-32485131-C-T	22	32485131	rs2057490468	C	T	Pathogenic	2733394	14	1614194	8.67E-06	FALSE
FBXO7	22-32487774-AGT-A	22	32487774	rs1157030138	AGT	A	Pathogenic	2900056	3	1609598	1.86E-06	FALSE
FBXO7	22-32491107-A-AT	22	32491107	rs2057531939	A	AT	Pathogenic	2960440	2	1613280	1.24E-06	FALSE
FBXO7	22-32493278-C-T	22	32493278	rs78099169	C	T	Pathogenic	2911981	8	1613536	4.96E-06	FALSE
FBXO7	22-32498174-G-T	22	32498174	rs1342038737	G	T	Pathogenic	1455741	14	1614112	8.67E-06	FALSE
FBXO7	22-32498177-TC-T	22	32498177	rs2057587984	TC	T	Pathogenic	2715290	18	1614048	1.12E-05	FALSE
FBXO7	22-32498410-TC-T	22	32498410	rs1344213916	TC	T	Pathogenic	2977139	3	1614172	1.86E-06	FALSE
FBXO7	22-32498453-C-T	22	32498453	rs121918304	C	T	Pathogenic	4809	16	1614170	9.91E-06	FALSE
GBA1	1-155235196-G-A	1	155235196	rs80356771	G	A	Pathogenic	4295	370	1612378	0.000229474726	TRUE
GBA1	1-155235680-C-T	1	155235680	rs1671699033	C	T	Pathogenic	1322984	7	1598222	4.38E-06	FALSE
GBA1	1-155235708-G-A	1	155235708		G	A	Pathogenic	1722541	2	1611508	1.24E-06	FALSE
GBA1	1-155235757-C-T	1	155235757	rs1553217009	C	T	Pathogenic	496081	8	1613982	4.96E-06	FALSE
GBA1	1-155235775-A-T	1	155235775	rs1557901552	A	T	Pathogenic	599275	1	1614214	6.19E-07	FALSE

gnomAD (v4.1.0) pathogenic variant information for Parkinson's Disease genes												
gene	gnomAD_ID	Chromosome	Position	rsIDs	Reference	Alternate	ClinVar_ClnSignificance	ClinVar_varID	allele_count	allele_number	allele_freq	in_gp2
GBA1	1-155235780-G-A	1	155235780	rs76910485	G	A	Pathogenic	931820	3	1614220	1.86E-06	FALSE
GBA1	1-155235810-C-T	1	155235810	rs1671711470	C	T	Pathogenic	918141	6	1614226	3.72E-06	FALSE
GBA1	1-155236277-G-A	1	155236277	rs121908309	G	A	Pathogenic	4326	22	1614110	1.36E-05	FALSE
GBA1	1-155236285-G-A	1	155236285	rs760307559	G	A	Pathogenic	918140	6	1614156	3.72E-06	FALSE
GBA1	1-155236295-G-A	1	155236295	rs121908308	G	A	Pathogenic	813336	11	1614020	6.82E-06	TRUE
GBA1	1-155236415-A-G	1	155236415		A	G	Pathogenic	2581173	1	1614166	6.20E-07	FALSE
GBA1	1-155236439-CA-C	1	155236439	rs1553217314	CA	C	Pathogenic	496079	4	1614182	2.48E-06	FALSE
GBA1	1-155237357-G-A	1	155237357	rs121908298	G	A	Pathogenic	4305	1	1613970	6.20E-07	FALSE
GBA1	1-155237370-G-A	1	155237370	rs765633380	G	A	Pathogenic	813337	30	1613904	1.86E-05	FALSE
GBA1	1-155237453-C-T	1	155237453	rs78973108	C	T	Pathogenic	4328	120	1613838	7.44E-05	TRUE
GBA1	1-155237454-G-A	1	155237454	rs1553217626	G	A	Pathogenic	558787	1	1613960	6.20E-07	FALSE
GBA1	1-155237520-C-T	1	155237520		C	T	Pathogenic	3064209	1	1614006	6.20E-07	FALSE
GBA1	1-155238174-C-T	1	155238174	rs409652	C	T	Pathogenic	93459	50	1614020	3.10E-05	FALSE
GBA1	1-155238194-C-T	1	155238194	rs74462743	C	T	Pathogenic	558788	13	1614154	8.05E-06	FALSE
GBA1	1-155238206-A-C	1	155238206	rs381427	A	C	Pathogenic	928835	9	1613832	5.58E-06	TRUE
GBA1	1-155238215-T-C	1	155238215	rs364897	T	C	Pathogenic	4314	100	1612400	6.20E-05	TRUE
GBA1	1-155238242-C-T	1	155238242	rs867929413	C	T	Pathogenic	632834	4	1613234	2.48E-06	FALSE
GBA1	1-155238260-G-C	1	155238260	rs1671872221	G	C	Pathogenic	996254	18	1609276	1.12E-05	FALSE
GBA1	1-155238597-G-A	1	155238597	rs398123530	G	A	Pathogenic	93453	22	1613446	1.36E-05	FALSE
GBA1	1-155238630-G-A	1	155238630	rs439898	G	A	Pathogenic	65570	43	1613512	2.66E-05	TRUE
GBA1	1-155239736-G-A	1	155239736	rs1671974195	G	A	Pathogenic	984479	1	1614058	6.20E-07	FALSE
GBA1	1-155239934-G-A	1	155239934	rs1141814	G	A	Pathogenic	4321	20	1614120	1.24E-05	TRUE
GBA1	1-155239937-G-A	1	155239937	rs1671987417	G	A	Pathogenic	1321421	7	1614050	4.34E-06	FALSE
GBA1	1-155239939-C-T	1	155239939	rs77829017	C	T	Pathogenic	4296	2	1614108	1.24E-06	FALSE
GBA1	1-155239989-CG-C	1	155239989	rs1170895261	CG	C	Pathogenic	1321450	1	1613966	6.20E-07	FALSE
GBA1	1-155240033-C-A	1	155240033	rs121908302	C	A	Pathogenic	4313	1	1614006	6.20E-07	FALSE
GBA1	1-155240637-C-T	1	155240637	rs777383151	C	T	Pathogenic	974986	1	1613372	6.20E-07	FALSE
GBA1	1-155240660-G-GC	1	155240660	rs387906315	G	GC	Pathogenic	4302	50	1613560	3.10E-05	FALSE
PLA2G6	22-38112253-CTG-C	22	38112253	rs587784352	CTG	C	Pathogenic	159764	9	1613590	5.58E-06	FALSE
PLA2G6	22-38112529-C-A	22	38112529	rs1296348337	C	A	Pathogenic	1012698	1	1555938	6.43E-07	FALSE
PLA2G6	22-38113522-CA-C	22	38113522	rs1281282603	CA	C	Pathogenic	3214186	2	1613966	1.24E-06	FALSE
PLA2G6	22-38113616-GACA-G	22	38113616	rs587784343	GACA	G	Pathogenic	6198	4	1613970	2.48E-06	FALSE
PLA2G6	22-38115579-G-A	22	38115579	rs767689496	G	A	Pathogenic	2724426	3	1613568	1.86E-06	FALSE
PLA2G6	22-38115587-G-C	22	38115587		G	C	Pathogenic	2170057	1	1613464	6.20E-07	FALSE
PLA2G6	22-38115592-C-T	22	38115592	rs1318351016	C	T	Pathogenic	1686076	2	1613272	1.24E-06	FALSE
PLA2G6	22-38115628-G-A	22	38115628	rs1484455290	G	A	Pathogenic	1028628	10	1607406	6.22E-06	FALSE
PLA2G6	22-38115658-G-A	22	38115658	rs587784339	G	A	Pathogenic	159749	46	1544374	2.98E-05	TRUE
PLA2G6	22-38115668-C-T	22	38115668	rs2145683127	C	T	Pathogenic	1180814	5	1600860	3.12E-06	FALSE
PLA2G6	22-38115679-G-A	22	38115679		G	A	Pathogenic	2748479	1	1601154	6.25E-07	FALSE
PLA2G6	22-38120835-C-A	22	38120835	rs777259654	C	A	Pathogenic	2862124	2	1613932	1.24E-06	FALSE
PLA2G6	22-38123161-T-TG	22	38123161	rs1172241523	T	TG	Pathogenic	2737044	10	1550552	6.45E-06	FALSE
PLA2G6	22-38123185-C-T	22	38123185	rs587784332	C	T	Pathogenic	379833	3	1552570	1.93E-06	FALSE
PLA2G6	22-38126369-A-G	22	38126369	rs1352483031	A	G	Pathogenic	2412648	1	1611342	6.21E-07	FALSE
PLA2G6	22-38126446-AG-A	22	38126446	rs587784329	AG	A	Pathogenic	159730	14	1612592	8.68E-06	FALSE
PLA2G6	22-38128354-GA-G	22	38128354	rs1282370486	GA	G	Pathogenic	652932	6	1613734	3.72E-06	FALSE

**gnomAD (v4.1.0) pathogenic variant information for Parkinson's Disease genes**												
gene	gnomAD_ID	Chromosome	Position	rsIDs	Reference	Alternate	ClinVar_ClnSignificance	ClinVar_varID	allele_count	allele_number	allele_freq	in_gp2
PLA2G6	22-38128402-CAG-C	22	38128402	rs1290554462	CAG	C	Pathogenic	2741274	4	1613996	2.48E-06	FALSE
PLA2G6	22-38129453-C-A	22	38129453	rs761815070	C	A	Pathogenic	2581775	4	1598064	2.50E-06	FALSE
PLA2G6	22-38129523-C-G	22	38129523	rs587784327	C	G	Pathogenic	2863736	2	1613964	1.24E-06	FALSE
PLA2G6	22-38132869-C-T	22	38132869	rs1569263730	C	T	Pathogenic	561083	4	1552272	2.58E-06	FALSE
PLA2G6	22-38132917-C-A	22	38132917	rs199935023	C	A	Pathogenic	30371	23	1557154	1.48E-05	TRUE
PLA2G6	22-38135061-A-C	22	38135061	rs587784362	A	C	Pathogenic	159780	1	1602272	6.24E-07	FALSE
PLA2G6	22-38140136-G-A	22	38140136	rs2088813126	G	A	Pathogenic	987078	1	1614122	6.20E-07	FALSE
PLA2G6	22-38143177-CATCT-C	22	38143177	rs2089023572	CATCT	C	Pathogenic	986119	9	1614080	5.58E-06	FALSE
PLA2G6	22-38169219-G-A	22	38169219	rs886039552	G	A	Pathogenic	265449	9	1611766	5.58E-06	FALSE
PLA2G6	22-38169300-G-A	22	38169300	rs761956614	G	A	Pathogenic	2412654	1	1614184	6.20E-07	FALSE
PLA2G6	22-38169318-G-A	22	38169318	rs200075782	G	A	Pathogenic	30370	58	1614026	3.59E-05	TRUE
PLA2G6	22-38169426-T-C	22	38169426	rs1167198937	T	C	Pathogenic	2412655	2	1613872	1.24E-06	FALSE
PINK1	1-20633561-C-T	1	20633561	rs1005937012	C	T	Pathogenic	661098	10	1182666	8.46E-06	FALSE
PINK1	1-20633622-CCGGCCGGGCCTACC	1	20633622	rs1480758482	CCGGCCGGGC	C	Pathogenic	1454290	3	1293628	2.32E-06	FALSE
PINK1	1-20633820-GC-G	1	20633820	rs755000580	GC	G	Pathogenic	664114	2	1577720	1.27E-06	FALSE
PINK1	1-20638052-GC-G	1	20638052	rs1557561340	GC	G	Pathogenic	581272	8	1614136	4.96E-06	FALSE
PINK1	1-20638073-CG-C	1	20638073	rs756677845	CG	C	Pathogenic	189240	2	1613932	1.24E-06	FALSE
PINK1	1-20639952-C-T	1	20639952	rs74315357	C	T	Pathogenic	2407	13	1612632	8.06E-06	FALSE
PINK1	1-20639990-C-A	1	20639990	rs756783990	C	A	Pathogenic	631591	3	1602136	1.87E-06	FALSE
PINK1	1-20645640-T-C	1	20645640	rs28940285	T	C	Pathogenic	2408	26	1614012	1.61E-05	TRUE
PINK1	1-20648994-G-A	1	20648994	rs2053228483	G	A	Pathogenic	934238	1	1612924	6.20E-07	FALSE
PINK1	1-20649054-G-A	1	20649054	rs74315356	G	A	Pathogenic	2406	8	1614040	4.96E-06	FALSE
PINK1	1-20649070-GC-G	1	20649070	rs775479526	GC	G	Pathogenic	1399771	30	1614094	1.86E-05	FALSE
PINK1	1-20649109-C-T	1	20649109	rs45539432	C	T	Pathogenic	2415	66	1614232	4.09E-05	TRUE
PINK1	1-20649217-C-T	1	20649217	rs34208370	C	T	Pathogenic	431963	101	1614054	6.26E-05	TRUE
PINK1	1-20649232-G-A	1	20649232	rs2053233432	G	A	Pathogenic	961640	4	1613880	2.48E-06	FALSE
PINK1	1-20650541-C-CCAA	1	20650541	rs750664040	C	CCAA	Pathogenic	2410	8	1614092	4.96E-06	FALSE
PARK7	1-7962867-C-T	1	7962867	rs374429170	C	T	Pathogenic	1399835	9	1612268	5.58E-06	FALSE
PARK7	1-7965336-G-GT	1	7965336	rs781600849	G	GT	Pathogenic	573287	32	1613906	1.98E-05	FALSE
PARK7	1-7965425-G-C	1	7965425	rs74315353	G	C	Pathogenic	7067	3	1613528	1.86E-06	FALSE
PARK7	1-7970963-G-A	1	7970963	rs1252815484	G	A	Pathogenic	1334459	2	1614192	1.24E-06	FALSE
LRRK2	12-40299125-A-G	12	40299125	rs34805604	A	G	Pathogenic	1939	2	1612972	1.24E-06	FALSE
LRRK2	12-40310434-C-G	12	40310434	rs33939927	C	G	Pathogenic	1936	5	1612956	3.10E-06	TRUE
LRRK2	12-40310434-C-T	12	40310434	rs33939927	C	T	Pathogenic	1938	39	1612956	2.42E-05	TRUE
LRRK2	12-40320129-C-T	12	40320129		C	T	Pathogenic	2572065	2	1611666	1.24E-06	FALSE
PRKN	6-161350163-C-T	6	161350163	rs961239925	C	T	Pathogenic	1459191	4	1613678	2.48E-06	FALSE
PRKN	6-161350214-G-C	6	161350214	rs765860776	G	C	Pathogenic	2136491	5	1608138	3.11E-06	TRUE
PRKN	6-161548965-GA-G	6	161548965	rs1562519380	GA	G	Pathogenic	1456905	8	1614124	4.96E-06	FALSE
PRKN	6-161569417-C-G	6	161569417	rs772074730	C	G	Pathogenic	1068478	2	1613248	1.24E-06	FALSE
PRKN	6-161785793-C-G	6	161785793	rs751037529	C	G	Pathogenic	409266	7	1614020	4.34E-06	TRUE
PRKN	6-161785839-A-T	6	161785839	rs377554392	A	T	Pathogenic	805244	13	1614148	8.05E-06	TRUE
PRKN	6-161973317-G-C	6	161973317	rs137853054	G	C	Pathogenic	7036	1	1610416	6.21E-07	FALSE
PRKN	6-161973401-C-T	6	161973401	rs137853058	C	T	Pathogenic	7046	7	1610136	4.35E-06	FALSE
PRKN	6-162054107-C-CT	6	162054107	rs1231455463	C	CT	Pathogenic	2174566	1	1609692	6.21E-07	FALSE
PRKN	6-162443314-A-T	6	162443314	rs137853059	A	T	Pathogenic	7047	33	1612238	2.05E-05	TRUE

gnomAD (v4.1.0) pathogenic variant information for Parkinson's Disease genes

gene	gnomAD_ID	Chromosome	Position	rsIDs	Reference	Alternate	ClinVar_ClnSignificance	ClinVar_varID	allele_count	allele_number	allele_freq	in_gp2
PRKN	6-162443325-AT-A	6	162443325	rs754809877	AT	A	Pathogenic	536457	449	1612394	0.000278467917	FALSE
PRKN	6-162443356-C-G	6	162443356	rs368134308	C	G	Pathogenic	644125	67	1613226	4.15E-05	TRUE
PRKN	6-162443378-CCT-C	6	162443378	rs55777503	CCT	C	Pathogenic	425403	543	1613910	0.000336449987	FALSE
PRKN	6-162443384-G-A	6	162443384	rs770591350	G	A	Pathogenic	853293	10	1613756	6.20E-06	FALSE
POLG	15-89317374-A-G	15	89317374	rs1335880349	A	G	Pathogenic	597808	6	1613830	3.72E-06	FALSE
POLG	15-89317375-C-T	15	89317375	rs1326779034	C	T	Pathogenic	2677947	1	1613940	6.20E-07	FALSE
POLG	15-89317389-G-GTATC	15	89317389	rs2055307723	G	GTATC	Pathogenic	2677952	8	1613984	4.96E-06	FALSE
POLG	15-89317448-TC-T	15	89317448		TC	T	Pathogenic	2024844	1	1614094	6.20E-07	FALSE
POLG	15-89317469-C-T	15	89317469	rs1131691575	C	T	Pathogenic	426100	4	1613976	2.48E-06	FALSE
POLG	15-89318540-C-A	15	89318540		C	A	Pathogenic	3013692	1	1612918	6.20E-07	FALSE
POLG	15-89318694-TGTAA-T	15	89318694	rs1442498340	TGTAA	T	Pathogenic	1457717	1	1614068	6.20E-07	FALSE
POLG	15-89318947-GAGGGCTCC-G	15	89318947		GAGGGCTCC	G	Pathogenic	2645687	1	1614088	6.20E-07	FALSE
POLG	15-89318963-G-A	15	89318963	rs767708989	G	A	Pathogenic	619310	5	1613954	3.10E-06	FALSE
POLG	15-89318986-G-A	15	89318986	rs267606959	G	A	Pathogenic	13516	18	1614078	1.12E-05	FALSE
POLG	15-89319044-CTG-C	15	89319044	rs1332921412	CTG	C	Pathogenic	817632	2	1614062	1.24E-06	FALSE
POLG	15-89319048-G-GC	15	89319048	rs1447799185	G	GC	Pathogenic	458711	2	1614028	1.24E-06	FALSE
POLG	15-89319225-T-A	15	89319225	rs778573169	T	A	Pathogenic	587863	31	1614058	1.92E-05	TRUE
POLG	15-89319229-TC-T	15	89319229	rs2055358006	TC	T	Pathogenic	872916	1	1614144	6.20E-07	FALSE
POLG	15-89319265-G-A	15	89319265	rs1567185770	G	A	Pathogenic	619307	1	1614180	6.20E-07	FALSE
POLG	15-89319274-TCCAGCCACCCTCA	15	89319274	rs886041276	TCCAGCCACCC	T	Pathogenic	279948	1	1614148	6.20E-07	FALSE
POLG	15-89319318-ACCAGCCACTCG-A	15	89319318	rs2055359441	ACCAGCCACTC	A	Pathogenic	1449732	1	1599762	6.25E-07	FALSE
POLG	15-89319349-AC-A	15	89319349	rs1314787391	AC	A	Pathogenic	2866014	1	1613918	6.20E-07	FALSE
POLG	15-89320883-T-C	15	89320883	rs113994099	T	C	Pathogenic	13495	1	1613974	6.20E-07	FALSE
POLG	15-89320919-C-T	15	89320919	rs1567186613	C	T	Pathogenic	619302	1	1613964	6.20E-07	FALSE
POLG	15-89320953-G-A	15	89320953	rs121918048	G	A	Pathogenic	13500	3	1614004	1.86E-06	FALSE
POLG	15-89321184-T-TC	15	89321184	rs1283198587	T	TC	Pathogenic	619306	4	1614062	2.48E-06	FALSE
POLG	15-89321242-C-A	15	89321242	rs121918047	C	A	Pathogenic	13504	3	1614188	1.86E-06	FALSE
POLG	15-89321743-T-C	15	89321743	rs121918050	T	C	Pathogenic	13506	3	1611710	1.86E-06	FALSE
POLG	15-89321780-G-A	15	89321780	rs144500145	G	A	Pathogenic	206528	179	1613984	0.000110905684	TRUE
POLG	15-89321783-T-C	15	89321783	rs775445970	T	C	Pathogenic	619395	5	1613898	3.10E-06	FALSE
POLG	15-89321792-C-T	15	89321792	rs113994098	C	T	Pathogenic	13502	498	1614136	0.000308524188	TRUE
POLG	15-89321961-C-T	15	89321961	rs1567187326	C	T	Pathogenic	619400	5	1614106	3.10E-06	FALSE
POLG	15-89321961-C-A	15	89321961		C	A	Pathogenic	2860133	1	1614106	6.20E-07	FALSE
POLG	15-89322748-C-G	15	89322748	rs796052887	C	G	Pathogenic	2736252	3	1613940	1.86E-06	FALSE
POLG	15-89322856-AG-A	15	89322856		AG	A	Pathogenic	2760375	1	1613918	6.20E-07	FALSE
POLG	15-89323452-G-T	15	89323452	rs750514687	G	T	Pathogenic	1390135	1	1614106	6.20E-07	FALSE
POLG	15-89323829-G-A	15	89323829	rs1254855971	G	A	Pathogenic	619398	4	1613492	2.48E-06	FALSE
POLG	15-89323847-G-A	15	89323847	rs867038717	G	A	Pathogenic	381519	4	1613918	2.48E-06	FALSE
POLG	15-89324138-AG-A	15	89324138	rs1567188632	AG	A	Pathogenic	619396	10	1614102	6.20E-06	FALSE
POLG	15-89325452-G-T	15	89325452	rs1465650547	G	T	Pathogenic	969298	2	1598360	1.25E-06	FALSE
POLG	15-89325520-G-A	15	89325520	rs121918046	G	A	Pathogenic	13499	7	1611770	4.34E-06	FALSE
POLG	15-89325539-C-T	15	89325539		C	T	Pathogenic	2748199	1	1613248	6.20E-07	FALSE
POLG	15-89325610-G-A	15	89325610	rs139717885	G	A	Pathogenic	381520	23	1613526	1.43E-05	TRUE
POLG	15-89325616-G-A	15	89325616	rs2152065937	G	A	Pathogenic	1421810	1	1613514	6.20E-07	FALSE
POLG	15-89325679-G-A	15	89325679	rs774474723	G	A	Pathogenic	1452045	13	1605794	8.10E-06	TRUE

gnomAD (v4.1.0) pathogenic variant information for Parkinson's Disease genes

gene	gnomAD_ID	Chromosome	Position	rsIDs	Reference	Alternate	ClinVar_ClnSignificance	ClinVar_varID	allele_count	allele_number	allele_freq	in_gp2
POLG	15-89326934-AG-A	15	89326934	rs1567191094	AG	A	Pathogenic	619448	1	1614088	6.20E-07	FALSE
POLG	15-89327040-C-T	15	89327040	rs2055531147	C	T	Pathogenic	1455434	1	1614216	6.19E-07	FALSE
POLG	15-89327046-C-T	15	89327046		C	T	Pathogenic	2810586	2	1614234	1.24E-06	FALSE
POLG	15-89327062-AC-A	15	89327062	rs2055531567	AC	A	Pathogenic	2843895	1	1614072	6.20E-07	FALSE
POLG	15-89327166-C-T	15	89327166	rs771623994	C	T	Pathogenic	280017	14	1614140	8.67E-06	TRUE
POLG	15-89327201-C-T	15	89327201	rs113994095	C	T	Pathogenic	13496	1637	1614256	0.00101408946	TRUE
POLG	15-89327244-A-C	15	89327244		A	C	Pathogenic	2677961	2	1614190	1.24E-06	FALSE
POLG	15-89327306-CA-C	15	89327306		CA	C	Pathogenic	2865640	1	1614050	6.20E-07	FALSE
POLG	15-89327311-A-G	15	89327311	rs1567191474	A	G	Pathogenic	619407	2	1613990	1.24E-06	FALSE
POLG	15-89327328-CAG-C	15	89327328	rs796052908	CAG	C	Pathogenic	206608	5	1613734	3.10E-06	FALSE
POLG	15-89327351-T-C	15	89327351	rs2055536585	T	C	Pathogenic	933883	1	1612794	6.20E-07	FALSE
POLG	15-89328735-G-A	15	89328735	rs960142425	G	A	Pathogenic	619394	4	1613996	2.48E-06	FALSE
POLG	15-89328781-CT-C	15	89328781	rs763418954	CT	C	Pathogenic	596511	12	1614042	7.43E-06	FALSE
POLG	15-89329041-G-A	15	89329041	rs886041592	G	A	Pathogenic	280375	7	1613060	4.34E-06	TRUE
POLG	15-89329059-C-T	15	89329059	rs749799663	C	T	Pathogenic	1425375	7	1612940	4.34E-06	FALSE
POLG	15-89330113-G-A	15	89330113	rs1057517803	G	A	Pathogenic	372472	5	1613966	3.10E-06	FALSE
POLG	15-89330231-C-T	15	89330231	rs1567192879	C	T	Pathogenic	619303	2	1613190	1.24E-06	FALSE
POLG	15-89330241-C-T	15	89330241	rs113994093	C	T	Pathogenic	21319	14	1612638	8.68E-06	FALSE
POLG	15-89333526-G-A	15	89333526		G	A	Pathogenic	2000637	1	1612882	6.20E-07	FALSE
POLG	15-89333553-G-A	15	89333553	rs202039305	G	A	Pathogenic	279988	3	1612020	1.86E-06	FALSE
POLG	15-89333619-G-A	15	89333619	rs2055628382	G	A	Pathogenic	2707771	1	1601374	6.24E-07	FALSE
POLG	15-89333680-C-T	15	89333680	rs1021719232	C	T	Pathogenic	694427	2	1548958	1.29E-06	FALSE
POLG	15-89333747-C-G	15	89333747	rs121918045	C	G	Pathogenic	13498	1	1535292	6.51E-07	FALSE
DNAJC6	1-65366107-C-T	1	65366107	rs864622011	C	T	Pathogenic	219301	3	1613868	1.86E-06	FALSE
DNAJC6	1-65385899-C-T	1	65385899	rs1645867120	C	T	Pathogenic	976692	4	1600054	2.50E-06	FALSE
SYNJ1	21-32638958-G-A	21	32638958	rs747261340	G	A	Pathogenic	1076901	11	1613954	6.82E-06	FALSE
SYNJ1	21-32643449-C-CT	21	32643449	rs2145756643	C	CT	Pathogenic	1070684	2	1613586	1.24E-06	FALSE
SYNJ1	21-32645791-T-C	21	32645791	rs1057524880	T	C	Pathogenic	393360	1	1482182	6.75E-07	FALSE
SYNJ1	21-32646432-G-A	21	32646432	rs1373545506	G	A	Pathogenic	978699	1	1614094	6.20E-07	FALSE
SYNJ1	21-32646513-TG-T	21	32646513	rs1230133310	TG	T	Pathogenic	848199	1	1613948	6.20E-07	FALSE
SYNJ1	21-32656686-CCTTAT-C	21	32656686	rs778394516	CCTTAT	C	Pathogenic	570902	5	1610262	3.11E-06	FALSE
SYNJ1	21-32665963-G-A	21	32665963		G	A	Pathogenic	1954917	5	1609376	3.11E-06	FALSE
SYNJ1	21-32678675-G-A	21	32678675		G	A	Pathogenic	2936446	1	1609890	6.21E-07	FALSE
SYNJ1	21-32685772-T-TA	21	32685772	rs1419316294	T	TA	Pathogenic	1074545	10	1606784	6.22E-06	FALSE
SYNJ1	21-32694269-G-A	21	32694269	rs1160469053	G	A	Pathogenic	1456091	4	1567130	2.55E-06	FALSE
SYNJ1	21-32695106-C-T	21	32695106	rs398122403	C	T	Pathogenic	88844	17	1614092	1.05E-05	FALSE
SYNJ1	21-32695131-G-A	21	32695131	rs756965178	G	A	Pathogenic	1323673	7	1613898	4.34E-06	FALSE
SYNJ1	21-32700028-G-A	21	32700028	rs2042344677	G	A	Pathogenic	1206765	4	1613928	2.48E-06	FALSE
SYNJ1	21-32726907-C-A	21	32726907	rs1040540690	C	A	Pathogenic	1457606	7	1613892	4.34E-06	TRUE
SYNJ1	21-32728027-C-CAT	21	32728027	rs1227986180	C	CAT	Pathogenic	1069960	1	1535332	6.51E-07	FALSE
SYNJ1	21-32728032-CT-C	21	32728032		CT	C	Pathogenic	2945224	1	1533782	6.52E-07	FALSE
VPS13C	15-61876978-C-A	15	61876978	rs751054856	C	A	Pathogenic	3006540	10	1599020	6.25E-06	FALSE
VPS13C	15-61878689-C-A	15	61878689	rs199723460	C	A	Pathogenic	1323753	1	1611370	6.21E-07	FALSE
VPS13C	15-61881570-G-A	15	61881570	rs1229922592	G	A	Pathogenic	2993124	5	1606698	3.11E-06	FALSE
VPS13C	15-61884244-GT-G	15	61884244	rs775841187	GT	G	Pathogenic	2662741	52	1610372	3.23E-05	FALSE

gnomAD (v4.1.0) pathogenic variant information for Parkinson's Disease genes

gene	gnomAD_ID	Chromosome	Position	rsIDs	Reference	Alternate	ClinVar_ClnSignificance	ClinVar_varID	allele_count	allele_number	allele_freq	in_gp2
VPS13C	15-61890331-G-A	15	61890331	rs1387456031	G	A	Pathogenic	2984894	7	1613896	4.34E-06	FALSE
VPS13C	15-61920161-A-AT	15	61920161	rs1315150327	A	AT	Pathogenic	1965239	7	1613480	4.34E-06	FALSE
VPS13C	15-61951850-A-AT	15	61951850		A	AT	Pathogenic	2822600	4	1611338	2.48E-06	FALSE
VPS13C	15-61961648-G-T	15	61961648	rs1180158172	G	T	Pathogenic	3238850	3	1613706	1.86E-06	FALSE
VPS13C	15-62023829-CT-C	15	62023829	rs1456557102	CT	C	Pathogenic	2724228	10	1610480	6.21E-06	FALSE
VPS13C	15-62033477-G-A	15	62033477	rs778239562	G	A	Pathogenic	1941006	34	1604990	2.12E-05	FALSE
VPS13C	15-62035047-AT-A	15	62035047	rs761323769	AT	A	Pathogenic	3000824	9	1602338	5.62E-06	FALSE
HTRA2	2-74530250-C-T	2	74530250	rs1675486182	C	T	Pathogenic	1424738	7	1609358	4.35E-06	FALSE
HTRA2	2-74532641-C-T	2	74532641	rs1407675367	C	T	Pathogenic	1455851	13	1613560	8.06E-06	FALSE
HTRA2	2-74532714-G-A	2	74532714	rs767006508	G	A	Pathogenic	372209	14	1613412	8.68E-06	FALSE
TMEM230	20-5100921-C-A	20	5100921	rs764786986	C	A	Pathogenic	243014	5	1613958	3.10E-06	TRUE
UCHL1	4-41256996-A-C	4	41256996	rs397515634	A	C	Pathogenic	88635	2	1614182	1.24E-06	FALSE
UCHL1	4-41257706-C-CGCT	4	41257706	rs749368841	C	CGCT	Pathogenic	2077519	3	1580822	1.90E-06	FALSE
UCHL1	4-41261751-CAG-C	4	41261751	rs1310363710	CAG	C	Pathogenic	3068443	1	1613326	6.20E-07	FALSE
UCHL1	4-41264108-C-T	4	41264108	rs2154087267	C	T	Pathogenic	1380167	1	1614208	6.19E-07	FALSE
