# Supplementary material for: Parkinson’s Disease Pathogenic Variants: Cross-Ancestry Analysis and Microarray Data Validation
Source: medRxiv. 2024 Dec 17:2024.12.16.24319097. Preprint. [Version 1] doi: 10.1101/2024.12.16.24319097 (PMC11702716; doi:10.1101/2024.12.16.24319097)
Supplement: Supplement 6 [file media-6.pdf]

All pathogenic variants per ancestry with freq (overall and case control)

			AAC			AFR			AJ			AMR			CAH			CAS			EAS			EUR			FIN			MDE			SAS		
GENE	rsID	snpID	MAF	MAF_A	MAF_U	MAF	MAF_A	MAF_U	MAF	MAF_A	MAF_U	MAF	MAF_A	MAF_U	MAF	MAF_A	MAF_U	MAF	MAF_A	MAF_U	MAF	MAF_A	MAF_U	MAF	MAF_A	MAF_U	MAF	MAF_A	MAF_U	MAF	MAF_A	MAF_U	MAF	MAF_A	MAF_U
LRRK2	rs33939927	chr12:40310434:C:T	NA	NA	NA	NA	NA	NA	NA	NA	NA	NA	NA	NA	NA	NA	NA	0.0005537	0.0009058	<1e-6	NA	NA	NA	0.0002189	0.0003303	5.43E-05	NA	NA	NA	0.003448	0.006452	<1e-6	NA	NA	NA
POLG	rs778573169	chr15:89319225:T:A	NA	NA	NA	NA	NA	NA	NA	NA	NA	NA	NA	NA	NA	NA	NA	NA	NA	NA	NA	NA	NA	3.86E-05	4.72E-05	<1e-6	NA	NA	NA	NA	NA	NA	NA	NA	NA
POLG	rs144500145	chr15:89321780:G:A	NA	NA	NA	NA	NA	NA	NA	NA	NA	NA	NA	NA	NA	NA	NA	NA	NA	NA	NA	NA	NA	9.01E-05	9.44E-05	0.0001628	NA	NA	NA	NA	NA	NA	NA	NA	NA
POLG	rs113994098	chr15:89321792:C:T	<1e-6	<1e-6	<1e-6	0.0001893	<1e-6	0.000298	NA	NA	NA	NA	NA	NA	NA	NA	NA	NA	NA	NA	NA	NA	NA	0.0002833	0.0002831	0.0002171	NA	NA	NA	NA	NA	NA	NA	NA	NA
POLG	rs139717885	chr15:89325610:G:A	NA	NA	NA	<1e-6	<1e-6	<1e-6	NA	NA	NA	NA	NA	NA	NA	NA	NA	NA	NA	NA	NA	NA	NA	NA	NA	NA	NA	NA	NA	NA	NA	NA	NA	NA	NA
POLG	rs774474723	chr15:89325679:G:A	NA	NA	NA	NA	NA	NA	NA	NA	NA	NA	NA	NA	NA	NA	NA	NA	NA	NA	NA	NA	NA	2.58E-05	<1e-6	5.43E-05	NA	NA	NA	NA	NA	NA	NA	NA	NA
POLG	rs771623994	chr15:89327166:C:T	NA	NA	NA	0.0001897	0.0005313	<1e-6	NA	NA	NA	NA	NA	NA	NA	NA	NA	NA	NA	NA	NA	NA	NA	NA	NA	NA	NA	NA	NA	NA	NA	NA	NA	NA	NA
POLG	rs113994095	chr15:89327201:C:T	0.00045	<1e-6	0.0006242	0.0001892	<1e-6	0.0002978	<1e-6	<1e-6	<1e-6	NA	NA	NA	NA	NA	NA	NA	NA	NA	NA	NA	NA	0.0007355	0.000804	0.0005438	NA	NA	NA	NA	NA	NA	NA	NA	NA
POLG	rs886041592	chr15:89329041:G:A	NA	NA	NA	NA	NA	NA	NA	NA	NA	NA	NA	NA	NA	NA	NA	NA	NA	NA	NA	NA	NA	2.58E-05	4.72E-05	<1e-6	NA	NA	NA	NA	NA	NA	NA	NA	NA
GBA	rs80356771	chr1:155235196:G:A	NA	NA	NA	NA	NA	NA	NA	NA	NA	NA	NA	NA	NA	NA	NA	NA	NA	NA	NA	NA	NA	0.0003479	0.0005904	0.0001086	NA	NA	NA	NA	NA	NA	NA	NA	NA
GBA	rs121908308	chr1:155236295:G:A	NA	NA	NA	NA	NA	NA	NA	NA	NA	NA	NA	NA	0.0005875	0.0009524	<1e-6	NA	NA	NA	NA	NA	NA	NA	NA	NA	NA	NA	NA	NA	NA	NA	NA	NA	NA
GBA	rs78973108	chr1:155237453:C:T	NA	NA	NA	NA	NA	NA	NA	NA	NA	NA	NA	NA	NA	NA	NA	NA	NA	NA	NA	NA	NA	0.000206	0.0002832	5.43E-05	NA	NA	NA	NA	NA	NA	NA	NA	NA
GBA	rs381427	chr1:155238206:A:C	NA	NA	NA	<1e-6	<1e-6	<1e-6	NA	NA	NA	NA	NA	NA	NA	NA	NA	NA	NA	NA	NA	NA	NA	NA	NA	NA	NA	NA	NA	NA	NA	NA	NA	NA	NA
GBA	rs364897	chr1:155238215:T:C	NA	NA	NA	0.0001892	0.0005308	<1e-6	NA	NA	NA	NA	NA	NA	NA	NA	NA	NA	NA	NA	NA	NA	NA	<1e-6	<1e-6	<1e-6	NA	NA	NA	NA	NA	NA	NA	NA	NA
GBA	rs439898	chr1:155238630:G:A	NA	NA	NA	NA	NA	NA	NA	NA	NA	NA	NA	NA	NA	NA	NA	NA	NA	NA	NA	NA	NA	6.44E-05	9.44E-05	<1e-6	NA	NA	NA	NA	NA	NA	NA	NA	NA
GBA	rs1141814	chr1:155239934:G:A	NA	NA	NA	NA	NA	NA	NA	NA	NA	NA	NA	NA	NA	NA	NA	NA	NA	NA	NA	NA	NA	NA	NA	NA	NA	NA	NA	0.001721	0.003215	<1e-6	NA	NA	NA
ATP13A2	rs144701072	chr1:16988455:C:T	NA	NA	NA	0.0001892	0.0005308	<1e-6	NA	NA	NA	NA	NA	NA	NA	NA	NA	NA	NA	NA	NA	NA	NA	NA	NA	NA	NA	NA	NA	NA	NA	NA	NA	NA	NA
ATP13A2	rs866035312	chr1:16989961:G:A	NA	NA	NA	NA	NA	NA	NA	NA	NA	<1e-6	<1e-6	<1e-6	NA	NA	NA	NA	NA	NA	NA	NA	NA	NA	NA	NA	NA	NA	NA	NA	NA	NA	NA	NA	NA
ATP13A2	rs773246271	chr1:16992345:G:A	NA	NA	NA	NA	NA	NA	NA	NA	NA	NA	NA	NA	NA	NA	NA	NA	NA	NA	NA	NA	NA	2.58E-05	4.72E-05	<1e-6	NA	NA	NA	NA	NA	NA	NA	NA	NA
ATP13A2	rs1303653650	chr1:16996059:G:A	NA	NA	NA	NA	NA	NA	NA	NA	NA	NA	NA	NA	NA	NA	NA	NA	NA	NA	0.0001935	0.0001878	0.0002032	<1e-6	<1e-6	<1e-6	NA	NA	NA	NA	NA	NA	NA	NA	NA
ATP13A2	rs373607247	chr1:17005450:C:T	NA	NA	NA	NA	NA	NA	NA	NA	NA	NA	NA	NA	NA	NA	NA	NA	NA	NA	NA	NA	NA	<1e-6	<1e-6	<1e-6	NA	NA	NA	NA	NA	NA	NA	NA	NA
PINK1	rs28940285	chr1:20645640:T:C	NA	NA	NA	NA	NA	NA	NA	NA	NA	NA	NA	NA	NA	NA	NA	NA	NA	NA	0.001941	0.003394	0.0004072	NA	NA	NA	NA	NA	NA	NA	NA	NA	NA	NA	NA
PINK1	rs45539432	chr1:20649109:C:T	NA	NA	NA	NA	NA	NA	NA	NA	NA	NA	NA	NA	NA	NA	NA	NA	NA	NA	NA	NA	NA	0.0002704	0.0003775	0.0001628	NA	NA	NA	0.0008606	0.001608	<1e-6	NA	NA	NA
PINK1	rs34208370	chr1:20649217:C:T	NA	NA	NA	NA	NA	NA	NA	NA	NA	NA	NA	NA	NA	NA	NA	NA	NA	NA	0.0005812	0.0007522	0.0004067	3.86E-05	2.36E-05	0.0001086	<1e-6	<1e-6	<1e-6	NA	NA	NA	NA	NA	NA
TMEM230	rs764786986	chr20:5100921:C:A	NA	NA	NA	NA	NA	NA	NA	NA	NA	NA	NA	NA	NA	NA	NA	NA	NA	NA	NA	NA	NA	<1e-6	<1e-6	<1e-6	NA	NA	NA	NA	NA	NA	NA	NA	NA
SYNJ1	rs1040540690	chr21:32726907:C:A	NA	NA	NA	0.0001899	0.0005336	<1e-6	NA	NA	NA	NA	NA	NA	NA	NA	NA	NA	NA	NA	NA	NA	NA	NA	NA	NA	NA	NA	NA	NA	NA	NA	NA	NA	NA
PLA2G6	rs587784339	chr22:38115658:G:A	NA	NA	NA	NA	NA	NA	NA	NA	NA	0.000774	<1e-6	0.003226	NA	NA	NA	NA	NA	NA	NA	NA	NA	2.58E-05	4.72E-05	<1e-6	NA	NA	NA	NA	NA	NA	NA	NA	NA
PLA2G6	rs199935023	chr22:38132917:C:A	NA	NA	NA	NA	NA	NA	NA	NA	NA	NA	NA	NA	NA	NA	NA	NA	NA	NA	0.0001937	0.0001881	0.0002033	NA	NA	NA	NA	NA	NA	NA	NA	NA	NA	NA	NA
PLA2G6	rs200075782	chr22:38169318:G:A	NA	NA	NA	NA	NA	NA	NA	NA	NA	NA	NA	NA	NA	NA	NA	0.0005537	0.0009058	<1e-6	NA	NA	NA	NA	NA	NA	NA	NA	NA	NA	NA	NA	NA	NA	NA
PRKN	rs765860776	chr6:161350214:G:C	NA	NA	NA	NA	NA	NA	NA	NA	NA	0.001548	0.002183	<1e-6	NA	NA	NA	NA	NA	NA	NA	NA	NA	NA	NA	NA	NA	NA	NA	NA	NA	NA	NA	NA	NA
PRKN	rs751037529	chr6:161785793:C:G	NA	NA	NA	NA	NA	NA	NA	NA	NA	NA	NA	NA	NA	NA	NA	NA	NA	NA	9.68E-05	<1e-6	0.0002033	NA	NA	NA	NA	NA	NA	NA	NA	NA	NA	NA	NA
PRKN	rs377554392	chr6:161785839:A:T	NA	NA	NA	NA	NA	NA	NA	NA	NA	NA	NA	NA	0.0005875	0.0009524	<1e-6	NA	NA	NA	NA	NA	NA	NA	NA	NA	NA	NA	NA	NA	NA	NA	NA	NA	NA
PRKN	rs137853059	chr6:162443314:A:T	NA	NA	NA	NA	NA	NA	0.0001883	0.000387	<1e-6	NA	NA	NA	NA	NA	NA	NA	NA	NA	NA	NA	NA	NA	NA	NA	NA	NA	NA	NA	NA	NA	NA	NA	NA
PRKN	rs368134308	chr6:162443356:C:G	NA	NA	NA	NA	NA	NA	NA	NA	NA	NA	NA	NA	NA	NA	NA	NA	NA	NA	NA	NA	NA	2.58E-05	4.72E-05	<1e-6	NA	NA	NA	NA	NA	NA	NA	NA	NA

MAF: minor allele frequency

MAF_A: minor allele frequency of individuals with Parkinson's Disease

MAF_U: minor allele frequency of control individuals
